# Inequality in prevalence of unmedicated hypertension or diabetes among older Filipinos: analysis of nationally representative survey data

**DOI:** 10.1016/j.ijcrp.2026.200617

**Published:** 2026-03-02

**Authors:** Aleli D. Kraft, Kayleen Gene R. Calicdan, Joseph J. Capuno, Mark Ryan B. Paguirigan, Christian Joy P. Cruz, Owen O'Donnell

**Affiliations:** a School of Economics, University of the Philippines Diliman, Philippines; b Population Institute, University of the Philippines Diliman, Philippines; c School of Economics and School of Health Policy and Management, Erasmus University Rotterdam, the Netherlands

**Keywords:** Blood pressure, Cardiovascular, Cardiometabolic, Medication, Adherence, User charges

## Abstract

**Background:**

Public clinics in the Philippines provide free anti-hypertensive and anti-diabetic medications, removing the price barrier to accessing effective treatments. We aimed to assess whether this is sufficient to eliminate inequality in prevalence of unmedicated hypertension (HTN) or diabetes (DM) among older Filipinos diagnosed with either condition.

**Methods:**

We used cross-sectional survey data from a sample representative of the Philippines’ population aged 60+ years in 2018-19. We selected participants who reported being diagnosed with HTN or DM. We estimated the probability of not taking medication for either condition (*unmedicated*) overall and by wealth index quintile, educational attainment and covariates. We used probit to estimate fully adjusted risk differences (RDs).

**Results:**

We estimated that 30.7% [95% CI: 26.1, 35.5] of older Filipinos diagnosed with HTN or DM were unmedicated. Age-sex adjusted prevalence was higher at lower wealth (poorest: 57.8%; 43.2, 71.5) and education (≤elementary: 34.5%; 29.5, 39.8). Prevalence was also higher for those who were: diagnosed with DM only, male, rural, living alone, not working, not receiving remittances, not senior citizen registered, cognitively impaired and smokers. With full adjustment, the poorest-richest quintile RD was 28.3 percentage points (pp) [13.6, 43.0] and the lowest-highest education RD was 4.2 pp [-6.9, 15.3].

**Conclusions:**

High and unequal risk of being unmedicated for diagnosed HTN or DM, despite free maintenance medications, suggests important non-price barriers related to transport, stockouts, awareness and adherence.

## Highlights

• Few studies examine the cascade of care for both hypertension and diabetes.
• We found 31% of older Filipinos diagnosed with either condition were unmedicated.
• Poorer and less educated groups were even less likely to be medicated.
• This inequality persists despite free provision of maintenance medications.
• This points to the importance of non-price barriers.

## 1 Introduction

Hypertension and diabetes frequently coexist [1,2], increase in prevalence with age [3,4] and are principal risk factors for cardiovascular disease [2,[5], [6], [7]]. They account for a large and rising share of the burden of disease in middle-income countries with ageing populations [[7], [8], [9]]. Yet, each condition, and the two together, can be managed cost-effectively, including in older populations, with medication as well as lifestyle modification [[10], [11], [12], [13], [14], [15]]. Prescription of anti-hypertensive and anti-diabetic medications is the core of evidence-based treatment guidelines [[16], [17], [18]].

Ongoing management of hypertension and diabetes with maintenance medications that are paid for out-of-pocket can impose a large, accumulating economic burden that drains household resources and jeopardizes living standards with catastrophic health payments [[19], [20], [21], [22]]. This can discourage adherence to prescribed medications, particularly among poorer patients, with resulting inequality in treatment and control of hypertension and diabetes [[23], [24], [25], [26], [27], [28], [29]].

In the Philippines, patients diagnosed with hypertension or diabetes can get prescribed first-line maintenance medications free from Hypertension and Diabetes Clubs (HDCs) at public clinics [30]. Those clinics are required to follow the PhilPEN protocol [31] – an adaptation of the World Health Organization Package of Essential Non-Communicable Disease Interventions [18] – for disease screening, diagnosis and management, and to fill prescriptions at monthly check-ups. Since poorer people make greater use of public clinics [32], HDCs can potentially reduce inequality in treatment of hypertension and diabetes.

Realization of this potential requires overcoming supply-side constraints that impede PhilPEN implementation [33], including medicine stockouts [34], as well as demand-side constraints that interfere with adherence to prescribed medication [35,36]. This study aimed to estimate inequality in the prevalence of unmedicated hypertension and/or diabetes among older Filipinos diagnosed with either or both conditions.

## 2 Methods

### 2.1 Data

We used data from the first wave of the Longitudinal Study on Ageing and Health in the Philippines (LSAHP), conducted from October 2018 to February 2019, that is representative of the population aged 60 years and older (60+) [37]. Stratification of provinces, and municipalities in the National Capital Region, was based on the (projected) proportion of the population aged 60+. There was systematic sampling of nine provinces and two municipalities, followed by probability-proportionate-to-size (of 60+ population) sampling of 167 barangays (communities), with implicit rural/urban stratification. Within each sampled barangay, a list of all residents aged 60+ was compiled and used as the sampling frame that was stratified into three age groups: 60-69, 70-79 and ≥ 80. The youngest group was selected proportional to its size in each sampled barangay, while the two older age groups were oversampled. This resulted in 5985 interviews, with 5510 conducted directly with an older person and the rest by proxy. The response rate was 94% [37].

### 2.2 Measurements

A participant was categorized as *diagnosed* if they reported having been diagnosed with hypertension, diabetes or both by giving at least one affirmative answer to two questions: “Have you been told by a doctor that you have high blood pressure/diabetes?” Among those who reported being diagnosed with each condition, we categorized a participant as unmedicated for that condition if they gave a negative answer to the respective question: “At present, do you take any medicines for hypertension/diabetes?” We categorized a participant as *unmedicated* if they were unmedicated for either condition. Those diagnosed with both conditions were considered unmedicated if they reported not taking medication for at least one of them.

We examined the propensity to be unmedicated by socioeconomic and demographic characteristics. We proxied economic status by a wealth index derived from principal components analysis of reported household assets, electricity access, internet access, house ownership, housing conditions and an indicator of enrolment in the conditional cash transfer programme (4Ps) for poor households with children (Supplementary Material (SM) Table S1) [38]. We used the index to categorize participants into *wealth quintile* groups (*poorest 20%*, …, *richest 20%*). We used self-reported *educational attainment* to distinguish three categories: no more than elementary schooling (*≤ elementary*), more than elementary but not college (*intermediate*) and college or higher (*≥ college*). *Sex* was reported as *female* or *male*. We categorized *age* into 5-year intervals, plus a top category (*60-64 years, 65-69 years, …, 80+ years*). We used the household roster to create four categories of *living arrangement*: *with children, alone, with spouse only* and *with others*. We used self-reported employment to distinguish between those currently *working* and not. We created a binary indicator (*welfare recipient*) of living in a household that reported being a beneficiary of the 4Ps program. Other binary indicators distinguished those living in households that reported receiving *remittance income* from relatives or friends and having *health insurance*. We created binary indicators of the participant reporting being a *registered senior citizen* and not having gone to a healthcare provider when feeling sick in the last six months (*forgone care*).

Using an Omron HEM-7120 digital monitor and standard procedure, blood pressure (BP) was measured three times (with a 1-min gap). We used the average from the last two readings to distinguish between three categories of *blood pressure*: *high* (systolic BP ≥ 140 OR diastolic BP ≥ 90), *normal* (systolic BP < 140 AND diastolic BP < 90) and *incomplete* (<3 BP measurements taken). We created a binary indicator (*limited ADL*) of a reported limitation in at least one Activity of Daily Living (ADL) on a standard instrument [39,40]. We categorized a participant as *cognitively impaired* if they made at least 3 (/10) errors on the Short Portable Mental Status Questionnaire [37,41]. We used self-reported *smoking* status to distinguish between *non-smoker, ex-smoker* and *current smoker*.

### 2.3 Statistical analysis

The analysis sample included participants categorized as *diagnosed* with hypertension, diabetes or both. With this sample, we estimated the percentage that was *unmedicated* overall and by the covariates described in the preceding sub-section. We adjusted for age and sex using the age-sex composition of the analysis sample as the reference. This involved i) estimating a probit model of being undiagnosed as a function of indicators of sex, age groups and the respective covariate, ii) using the estimates to predict the probability each participant would be undiagnosed if they were in one category of the covariate, and iii) averaging those predictions across the analysis sample. We tested equal prevalence across categories of each covariate with a z-test of equal proportions for 2-category covariates and a chi-squared test of independence for other covariates.

We estimated the fully adjusted difference in the risk of being undiagnosed between each category and a reference category of each covariate. This was done by i) estimating a probit model of being undiagnosed as a function of all the covariates, plus indicators of province/municipality, ii) using the estimates to predict, for each participant, the probability of being undiagnosed if they were in a covariate category and if they were in the reference category for that covariate, iii) taking the difference in these predictions, and iv) averaging the differences over all participants to get the estimated population-averaged risk difference (RD). For estimation of RDs, as opposed to risk ratios, the advantage of probit over log-binomial and modified Poisson regressions is direct estimation of probabilities restricted to the 0-1 interval.

We conducted two supplementary analyses. First, we estimated the prevalence of diagnosed hypertension and/or diabetes by covariates, with age-sex adjustment. Second, we further restricted the sample to participants with diagnosed hypertension and/or diabetes and with high BP, and we estimated unmedicated RDs to identify covariate categories associated with being unmedicated and having high BP.

Sample weights were applied in all analyses. 95% confidence intervals were reported and adjusted for sample stratification and cluster sampling. All analyses were conducted using Stata version 18. The study was reported in accordance with Strengthening the Reporting of Observational Studies in Epidemiology (STROBE) guidelines (**SM Checklist S1**).

## 3 Results

**Fig. 1:**
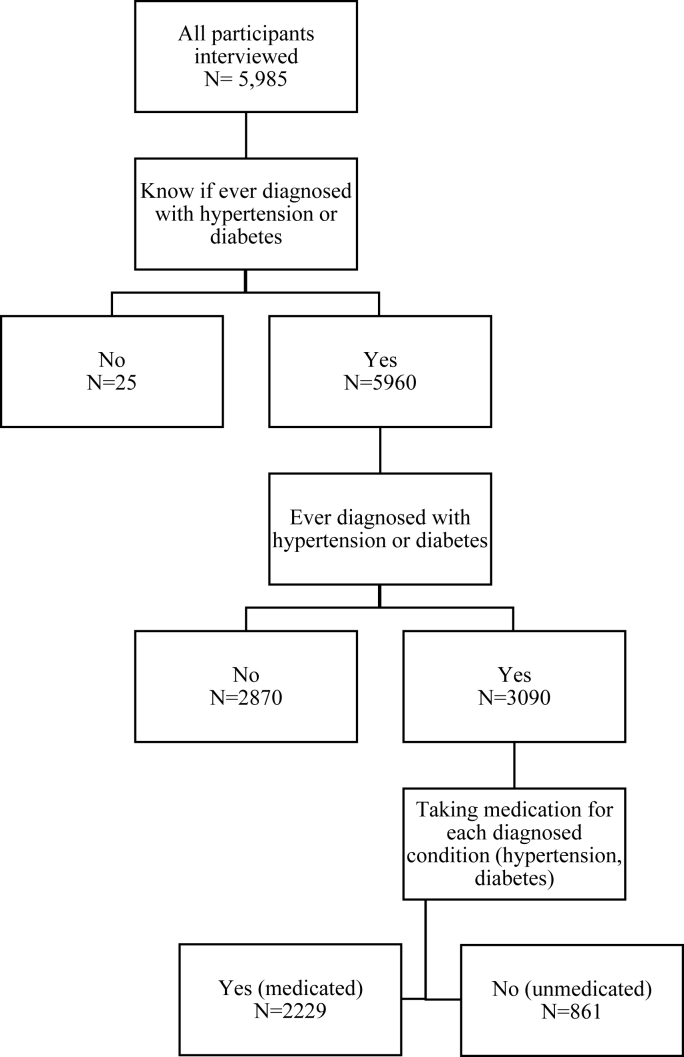
Participant flow.

Out of the total sample of 5985 participants interviewed (SM Table S2), 5960 reported knowing whether they had ever been diagnosed with hypertension and/or diabetes, of whom 3090 reported that they had been diagnosed with hypertension and/or diabetes (Fig. 1). We estimated that 48.9% [95% CI: 45.4, 52.3] of older Filipinos aged 60+ were diagnosed with either or both conditions. Prevalence was higher among individuals who were wealthier, more educated, female, urban, not living alone, not working, not a welfare recipient, registered as a senior citizen, limited in ADLs, not cognitively impaired and non-smokers (SM Table S3).

Table 1 shows characteristics of the analysis sample of 3090 participants who reported having been diagnosed with hypertension and/or diabetes. Around three quarters reported being diagnosed with hypertension only, less than one fifth were diagnosed with both conditions and around 7% were diagnosed with diabetes only. The analysis sample differs from the full sample in covariates associated with diagnosis (SM Table S3). For example, only about 14% of the analysis sample is in the poorest of the quintile group, which was defined to have (weighted) 20% of the full sample. Around two thirds of analysis sample participants have no more than elementary education, two thirds are female, three fifths are aged 60-69 years and just over one half (52.6%) are rural dwellers. Almost three fifths live with their children, three fifths are not working, over 90% are registered as senior citizens and around four fifths reported having health insurance.

**Table 1:** Characteristics of analysis sample, aged 60+ years and diagnosed with hypertension or diabetes.

	N	(%)
Overall	3090	(100.0)
Diagnosis		
Hypertension only	2380	(74.2)
Diabetes only	220	(6.9)
Both	490	(18.9)
Wealth quintile		
Poorest	401	(14.0)
Poorer	598	(17.6)
Middle	665	(20.1)
Richer	731	(25.0)
Richest	695	(23.3)
Educational attainment		
≤ Elementary	1981	(67.6)
Intermediate	755	(22.0)
≥ College	354	(10.4)
Sex		
Females	2107	(64.8)
Males	983	(35.2)
Age		
60-64 years	573	(35.7)
65-69 years	483	(24.4)
70-74 years	755	(16.4)
75-79 years	542	(11.7)
80+ years	737	(11.8)
Location		
Urban	1425	(47.4)
Rural	1665	(52.6)
Living arrangement		
with children	1890	(58.9)
Alone	379	(11.3)
with spouse only	285	(10.5)
with other	536	(19.3)
Working		
Yes	841	(38.7)
No	2249	(61.3)
Welfare recipient		
Yes	348	(10.2)
No	2742	(89.8)
Remittance income		
Yes	2360	(70.3)
No	730	(29.7)
Health insurance		
Yes	2410	(80.2)
No	680	(19.8)
Registered senior citizen		
Yes	2824	(91.9)
No	266	(8.1)
Forgone care		
Yes	883	(27.8)
No	2207	(72.2)
Blood pressure		
High (SBP ≥140 OR DBP ≥90)	1827	(59.0)
Normal (SBP <140 AND DBP <90)	1047	(35.6)
Incomplete	216	(5.4)
Limited ADL		
Yes	855	(27.4)
No	2235	(72.6)
Cognitively impaired		
Yes	382	(9.8)
No	2708	(90.2)
Smoking		
Non-smoker	1977	(62.6)
Ex-smoker	803	(25.5)
Current smoker	310	(11.9)

*Note*. N unweighted, % weighted. ADL = Activities of Daily Living. SBP = systolic blood pressure, DBP = diastolic blood pressure.

Table 2 shows estimated percentages of older Filipinos diagnosed with hypertension and/or diabetes that are unmedicated for either or both conditions. Overall, we estimated that 30.7% [26.1, 35.5] of those diagnosed are unmedicated. The point estimates indicate that, in the sample, those diagnosed with diabetes only are about 12 percentage points (pp) more likely to be unmedicated than those diagnosed with hypertension (without or with diabetes), although equal prevalence by type of diagnosis is not rejected (P = 0.508).

**Table 2:** Unmedicated percentage among older (60+) Filipinos diagnosed with hypertension and/or diabetes by covariates, age-sex adjusted (N = 3090).

	%	(95% CI)		P
Overall	30.7	(26.1,	35.5)	
Diagnosis				0.508
Hypertension only	29.8	(25.1,	34.9)	
Diabetes only	41.7	(21.9,	63.9)	
Both	30.2	(19.4,	43.0)	
Wealth quintile				<0.001
Poorest	57.8	(43.2,	71.5)	
Poorer	35.7	(27.8,	44.4)	
Middle	33.9	(26.5,	42.1)	
Richer	25.6	(16.1,	37.5)	
Richest	13.2	(9.0,	18.7)	
Education				0.031
≤ Elementary	34.5	(29.5,	39.8)	
Intermediate	23.7	(16.0,	33.0)	
≥ College	20.6	(7.0,	43.5)	
Sex				0.101
Females	27.9	(23.2,	33.0)	
Males	36.0	(27.3,	45.5)	
Age				0.741
60-64 years	30.9	(21.3,	42.1)	
65-69 years	33.1	(23.0,	44.6)	
70-74 years	26.2	(19.2,	34.2)	
75-79 years	35.6	(22.6,	50.6)	
80+ years	26.7	(20.2,	34.2)	
Location				0.019
Urban	24.3	(17.1,	32.8)	
Rural	36.5	(31.1,	42.0)	
Living arrangement				0.184
with children	28.1	(22.1,	34.8)	
Alone	44.5	(28.5,	61.5)	
with spouse only	33.2	(21.4,	46.9)	
with others	29.0	(20.4,	39.1)	
Working				0.071
Yes	26.1	(20.0,	33.0)	
No	38.1	(28.9,	48.0)	
Welfare recipient				0.286
Yes	30.0	(25.0,	35.4)	
No	36.6	(26.3,	48.0)	
Remittance income				0.016
Yes	41.3	(29.9,	53.4)	
No	26.2	(22.2,	30.6)	
Health insurance				0.161
Yes	37.9	(27.6,	49.2)	
No	28.9	(23.7,	34.6)	
Registered Senior Citizen				<0.001
No	55.0	(40.1,	69.2)	
Yes	28.5	(24.1,	33.3)	
Forgone care				0.209
Yes	35.4	(27.9,	43.5)	
No	28.8	(23.1,	35.2)	
Blood pressure				0.482
High (SBP ≥140 OR DBP ≥90)	28.8	(18.8,	40.8)	
Normal (SBP <140 AND DBP <90)	32.3	(26.2,	39.0)	
Incomplete	24.5	(14.9,	36.5)	
Limited ADL				0.718
Yes	29.7	(22.5,	37.8)	
No	31.0	(26.4,	36.0)	
Cognitively impaired				0.036
Yes	47.6	(29.8,	65.9)	
No	28.8	(24.6,	33.4)	
Smoking				0.058
Non-smoker	25.8	(20.1,	32.3)	
Ex-smoker	36.7	(29.2,	44.7)	
Current smoker	44.2	(28.7,	60.7)	

*Note*. P (values) from z tests of equal proportions between 2 groups and chi-square tests of independence for >2 groups. ADL = Activities of Daily Living. SBP = systolic blood pressure, DBP = diastolic blood pressure. By sex is adjusted for age. By age is adjusted for sex. All else are adjusted for age and sex.

Propensity to be unmedicated decreases steeply with increasing wealth (P < 0.001): an estimated 57.8% [43.2, 71.5] of those in the poorest fifth of older Filipinos and who were diagnosed with either or both conditions are unmedicated, compared with 13.2% [9.0, 18.7] of the richest fifth. The least educated were estimated to be about 14 pp more likely to be unmedicated than the most educated (P = 0.031). The estimated probability of a male being unmedicated is about 8 pp higher than it is for a female (P = 0.101). There is no systematic association with age. Rural dwellers are about 12.2 pp more likely to be unmediated than those in urban locations (P = 0.019). Those living alone are 11.3 pp more likely to be unmedicated than the living arrangement with the next highest prevalence (living with spouse only), although equal prevalence across all these groups is not rejected (P = 0.184). The probability of being unmedicated is higher for those who are not working (P = 0.071), are receiving remittances (P = 0.016) and are not registered as a senior citizen (P < 0.011). In the sample, those who reported having forgone care when sick are 6.6 pp more likely to be unmedicated, although no difference by the characteristic is not rejected (P = 0.209). The probability of being unmedicated does not vary across BP categories (P = 0.482) nor with having limited ADL (P = 0.718), but it is higher for the cognitively impaired (P = 0.036) and current smokers (P = 0.058).

Table 3 shows the estimated fully adjusted RDs for being unmedicated. With adjustment for all covariates, we estimated that those diagnosed with diabetes only are 22.7 pp [6.5, 38.8] more likely to be unmedicated than those diagnosed with hypertension only. After full adjustment, the wealth gradient in the propensity to be unmedicated remained clear – the poorest fifth were estimated to be 28.3 pp [13.6, 43.0] more likely to go unmedicated than the richest fifth. The education gradient did not remain after controlling for all covariates. With this control, we estimated that the risk of being unmedicated is 14.2 pp [3.8, 24.6] higher for those with no remittances than for those with such income, 13.2 pp [2.3, 24.0] higher for those not registered as a senior citizen, 13.7 pp [−2.6, 29.9] higher for the cognitively impaired and 12.7 pp [0.6, 24.7] higher for current smokers. After full adjustment, we found no differences in the probability of being unmedicated by sex, location, living arrangement and work status.

**Table 3:** Unmedicated risk difference (RD) by covariates (percentage points, pp), older (60+) Filipinos diagnosed with hypertension or diabetes (N = 3090).

	RD (pp)	(95% CI)	P
Diagnosis (ref. hypertension only)			
Diabetes only	22.7	(6.5,38.8)	0.006
Both HTN and diabetes	6.6	(-2.4,15.6)	0.151
Wealth quintile (ref. Richest)			
Poorest	28.3	(13.6,43.0)	<0.001
Poorer	12.5	(1.0,24.0)	0.033
Middle	13.0	(3.1,22.8)	0.010
Richer	10.8	(3.0,18.6)	0.007
Educational attainment (ref. ≥ College)			
≤ Elementary	4.2	(-6.9,15.3)	0.459
Intermediate	3.2	(-10.7,17.2)	0.650
Sex (ref. male)			
Females	0.7	(-9.3,10.6)	0.897
Age (ref. 80+)			
60-64 years	−0.3	(-9.4,8.9)	0.951
65-69 years	3.1	(-10.2,16.3)	0.649
70-74 years	−3.0	(-12.9,6.8)	0.544
75-79 years	8.3	(-7.0,23.5)	0.286
Location (ref. Urban)			
Rural	1.0	(-8.4,10.5)	0.830
Living (ref. with others)			
with children	2.5	(-6.6,11.5)	0.588
Alone	1.3	(-11.7,14.2)	0.849
with spouse only	−0.4	(-13.1,12.2)	0.948
Working (ref. No)			
Currently working	6.8	(-2.0,15.6)	0.129
Welfare recipient (ref. Yes)			
No	5.0	(-4.8,14.7)	0.318
Remittance income (ref. Yes)			
No	14.2	(3.8,24.6)	0.008
Health insurance (ref. Yes)			
No	5.4	(-3.3,14.0)	0.223
Senior citizen registration (ref. Yes)			
No	13.2	(2.3,24.0)	0.018
Forgone care (ref. No)			
Yes	3.3	(-3.8,10.4)	0.356
Blood pressure (ref. Normal)			
High (SBP ≥140 OR ≥ DBP 90)	3.5	(-6.0,13.0)	0.465
Incomplete	0.0	(-14.0,14.1)	0.996
Limited ADL (ref. No)			
Yes	−0.4	(-6.5,5.7)	0.900
Cognitively impaired (ref. No)			
Yes	13.7	(-2.6,29.9)	0.099
Smoking (ref. Non-smoker)			
Ex-smoker	8.3	(-0.2,16.9)	0.055
Current smoker	12.7	(0.6,24.7)	0.040

Note. Probit model estimates of difference in risk of being unmedicated compared with reference category in percentage points (pp). Province/municipality fixed effects also included in model. P (values) for test of H_0_: RD = 0.

Among those diagnosed with hypertension and/or diabetes and who had high BP, we estimated that the fully adjusted risk of being unmedicated is higher by 23.7 pp [8.3, 39.1] for those in the poorest fifth compared with the richest fifth, by 10 pp for the lower education groups compared with the most educated and by about 25 pp for those not registered as senior citizens compared with those who are (SM Table S4).

## 4 Discussion

Our finding that almost one third of older Filipinos diagnosed with hypertension and/or diabetes are unmedicated for either or both conditions indicates a very large gap in management of principal risk factors for CVD. It adds to evidence, obtained from the same dataset, that more than half of older Filipinos with hypertension (diagnosed or undiagnosed) are unmedicated [25]. We showed that the risk of being unmedicated is high even among those who are *diagnosed* with hypertension, and it is even higher (more than 40%) among those diagnosed with diabetes only. Given the complications and risks associated with uncontrolled diabetes, such a large proportion of those diagnosed with the condition going unmedicated is of even greater concern than the gap in hypertension management. Our estimate that around 1 in 8 older Filipinos are diagnosed with diabetes (SM Table S3) further intensifies the concern. Unfortunately, it is not restricted to the Philippines. Worldwide, an estimated 59% of those with diabetes (not necessarily diagnosed) are unmedicated, with Southeast Asia among the regions where treatment coverage remains stagnant while prevalence is rising rapidly [42]. Almost one third of those diagnosed with both hypertension and diabetes are unmedicated, which leaves those older people exposed to high CVD risk.

The treatment gap we identify exists despite the foundation of Hypertension and Diabetes Clubs (HDCs) in 2016, with the aim of providing free maintenance medications (thiazide diuretics, beta blockers, angiotensin converting enzyme inhibitors, calcium channel blockers, aspirin, metformin, glibenclamide and gliclazide). The HDCs operate in rural health units and public health clinics that poor older Filipinos utilize [32]. Nonetheless, we find a very steep wealth gradient in unmedicated (diagnosed) hypertension and/or diabetes to the disadvantage of the poor. Several factors may weaken the effectiveness of the HDCs in reducing this inequality.

First, not all poor older Filipinos may have easy access to the HDCs. Among those with hypertension (diagnosed and undiagnosed), there is a steep pro-rich wealth gradient also in the utilization of health facilities [43]. This may partly be due to limited access: half of the population must travel for more than 30 min to reach a public clinic [44]. We found that those living in rural locations were substantially more likely to be unmedicated.

Second, even if poor older people can reach clinics, they may find that medications are not available due to frequent stockouts [34]. A randomized experiment in one province found that visiting a public clinic increased the likelihood of having blood pressure measured but did not increase the likelihood of taking antihypertensives, despite a high prevalence of hypertension [33].

Third, many poor older Filipinos may be unaware that HDCs provide free medication. We found that around one fifth of older persons diagnosed with hypertension or diabetes reported not having health insurance despite all people aged 60+ being automatically covered by tax-financed national health insurance. About 8% of our analysis sample reported not being registered as a senior citizen despite age (60+) being the only criterion to qualify and registration giving entitlement to discounts on (non-HDC) medication charges. The unregistered were much more likely to be unmedicated, although this cross-sectional association need not be causal. Lack of awareness of HDCs and other coverage may leave many believing they face substantial financial barriers to medicating their condition. Almost 30% of our sample reported having forgone care when sick in the last six months, with cost the most cited reason. Our finding that those receiving remittance income are less likely to be unmedicated is consistent with (but does not imply) the existence of residual financial constraints on medication. While HDCs provide first-line medications for hypertension and diabetes, the benefit package includes only two oral anti-diabetics, only one of which (metformin) is usually available for free in public clinics. Patients must pay out-of-pocket for other oral anti-diabetics and insulin. Along with supply-side constraints on the availability of insulin (and injection devices) and non-price barriers to accessing stocks that are supplied [45,46], this limited coverage of essential medicines may contribute to our finding that a very high proportion of older Filipinos with diagnosed diabetes that are unmedicated.

Fourth, even when medication is available and affordable, behavioural biases can reduce adherence. Poverty is associated with less cognitive bandwidth for decisions, such as whether to adhere to maintenance medication, with consequences that are significant for long-term wellbeing but are less salient than immediate challenges confronted at, and below, the poverty threshold [47,48]. Our finding that the cognitively impaired are more likely to be unmedicated is consistent with this explanation, although it certainly does not confirm it.

The diversity of these hypothesized explanations for inequality in the prevalence of unmedicated hypertension and/or diabetes points toward the potential for a multi-pronged strategy to reduce it. Access to free maintenance medications most likely needs to improve, as does reliable supply of them, but demand-side interventions are also required to make poor older Filipinos more aware of the risks that can arise from their chronic conditions, the effectiveness of available medications in reducing these risks and their entitlements to get that medication without charge or with reduced charges.

There are several study limitations. First, diagnosis and medication were self-reported. Those falsely reporting having been diagnosed will likely be misclassified as unmedicated, increasing estimated prevalence. On the other hand, those forgetting they were diagnosed and, presumably, not taking (nor reporting) medication will cause bias in the opposite direction. While it is likely that the cognitively impaired make more reporting errors, it is unlikely that this explains why they are estimated to be at substantially greater risk of being unmedicated. Second, we focused on treatment through medication and, due to data constraints, ignored medical advice on lifestyle modification. Among those told by a doctor that they have high blood pressure, some may have been advised to change their diet and do more physical exercise without being prescribed medication. Some we identified as unmedicated may, therefore, have been appropriately treated. Third, we did not examine consequences of unmedicated hypertension and/or diabetes measured by uncontrolled blood pressure and blood glucose, respectively. Related, model inclusion of high blood pressure, which may be due to not taking medication, could result in overadjustment of unmedicated risk differences by covariates. However, among those with high blood pressure, who are at greatest risk, we still found differences in the probability of being unmedicated by wealth, education and senior citizen registration. Fourth, we relied on a binary indicator of medication because there were no data on frequency of medications. Among those who reported taking medication, there will be some who did not follow the prescription instructions. Fifth, the data do not provide information on the duration for which a diagnosed condition has gone unmedicated. Sixth, while the cross-sectional analysis reveals that unmedicated hypertension and/or diabetes is prevalent and unequally distributed among older Filipinos, it does not identify causes of the level nor the inequality. Finally, while hypertension and diabetes are clinically distinct conditions with different treatment pathways and access barriers, we estimated the proportion unmedicated for one or both diagnosed conditions, which may introduce clinically relevant misclassification. We aggregated the conditions because HDCs provide free maintenance medications to those diagnosed with either or both of them, and so there is interest in the proportion remaining unmedicated in that target population. We did estimate differences between the conditions in the proportion unmedicated.

A large proportion of older Filipinos diagnosed with hypertension and/or diabetes, particularly the poorer ones, go without pharmacotherapy. This finding underscores the need to strengthen Hypertension and Diabetes Clubs in the Philippines, and it demonstrates that granting entitlement to free maintenance medication is not sufficient to reduce substantial inequalities in management of diagnosed chronic conditions.

## CRediT authorship contribution statement

**Aleli D. Kraft:** Writing – review & editing, Writing – original draft, Supervision, Project administration, Funding acquisition. **Kayleen Gene R. Calicdan:** Visualization, Software, Data curation. **Joseph J. Capuno:** Writing – review & editing, Writing – original draft, Supervision, Resources, Project administration, Funding acquisition. **Mark Ryan B. Paguirigan:** Validation, Data curation, Conceptualization. **Christian Joy P. Cruz:** Validation, Data curation, Conceptualization. **Owen O'Donnell:** Writing – review & editing, Writing – original draft, Validation, Supervision, Project administration, Methodology, Funding acquisition, Formal analysis, Conceptualization.

## Financial interests statement

The collection of the baseline data of the Longitudinal Study of Ageing and Health in the Philippines was funded by the Economic Research Institute for ASEAN and East Asia. The study was funded by the Swiss Agency for Development and Cooperation/National Science Foundation Programme for Research on Global Issues for Development, Grant 400640_160374 (PI: Jurgen Maurer). The funders played no role in the conception, execution or reporting of the research presented in this article. The authors have no financial interests to declare.

## Data sharing agreement

The data from this study are available upon reasonable request here https://www.drdf.org.ph/lsahp-baseline-data-request-portal/.

## Ethical approval

The LSAHP survey was approved by the University of the Philippines Manila Research Ethics Board Review Panel 2. Written informed consent was obtained from all respondents. No further ethical approval was required for the secondary analysis of LSAHP data conducted for this study.

## Funding

This research was support by grants from the Economic Research Institute for ASEAN and East Asia, and the Swiss Agency for Development and Cooperation/Swiss National Science Foundation grant 400640_160374. The funders had no role in the study design, its conduct, the interpretation and reporting of results, preparation of the manuscript and its submission for publication.

## Declaration competing interest

The authors declare that they have no competing interests.

## References

[1] Bretzel R.G. (2007 Jan). Comorbidity of diabetes mellitus and hypertension in the clinical setting: a review of prevalence, pathophysiology, and treatment perspectives. Clin. Ther..

[2] Sunkara N., Ahsan H.C. (2017 Mar). Hypertension in diabetes and the risk of cardiovascular disease. Cardiovasc Endocrinol.

[3] Staessen J.A., Wang J., Bianchi G., Birkenhäger W.H. (2003 May). Essential hypertension. Lancet.

[4] Kirkman M.S., Briscoe V.J., Clark N., Florez H., Haas L.B., Halter J.B. (2012 Nov 14). Diabetes in older adults. Diabetes Care.

[5] The Emerging Risk Factors Collaboration (ERFC) (2010 Jun). Diabetes mellitus, fasting blood glucose concentration, and risk of vascular disease: a collaborative meta-analysis of 102 prospective studies. Lancet.

[6] Yen F.-S., Wei J.C.-C., Chiu L.-T., Hsu C.-C., Hwu C.-M. (2022 Jan 3). Diabetes, hypertension, and cardiovascular disease development. J. Transl. Med..

[7] Wu S., Xu W., Guan C., Lv M., Jiang S., Jinhua Z. (2023 May). Global burden of cardiovascular disease attributable to metabolic risk factors, 1990–2019: an analysis of observational data from a 2019 global burden of disease study. BMJ Open.

[8] Mensah G.A., Fuster V., Murray C.J.L., Roth G.A. (2023). Global burden of cardiovascular diseases and risks, 1990-2022. J. Am. Coll. Cardiol..

[9] Goh R.S.J., Chong B., Jayabaskaran J., Jauhari S.M., Chan S.P., Kueh M.T.W. (2024). The burden of cardiovascular disease in Asia from 2025 to 2050: a forecast analysis for East Asia, South Asia, South-East Asia, Central Asia, and high-income Asia Pacific regions. Lancet Reg. Health West. Pac..

[10] Hanna I.R., Wenger N.K. (2005). Secondary prevention of coronary heart disease in elderly patients. Am. Fam. Physician.

[11] Lim S.S., Gaziano T.A., Gakidou E., Reddy K.S., Farzadfar F., Lozano R. (2007 Dec). Prevention of cardiovascular disease in high-risk individuals in low-income and middle-income countries: health effects and costs. Lancet.

[12] Fleg J.L., Forman D.E., Berra K., Bittner V., Blumenthal J.A., Chen M.A. (2013 Nov 26). Secondary prevention of atherosclerotic cardiovascular disease in older adults. Circ..

[13] Foguet-Boreu Q., Violán C., López Jiménez T., Pons-Vigués M., Rodríguez-Blanco T., Valderas J.M. (2017 Aug). Pharmacological control of diabetes and hypertension comorbidity in the elderly: a study of “Real world” data. Prim. Care Diabetes.

[14] World Health Organization (WHO) (2017). https://www.who.int/publications/i/item/WHO-NMH-NVI-17.9.

[15] Harmand M.G.-C., del Mar García-Sanz M., Agustí A., Prada-Arrondo P.C., Domínguez-Rodríguez A., Grandal-Leirós B. (2022 Nov 28). Review on the management of cardiovascular risk factors in the elderly. J. Geriatric Cardiol..

[16] American Diabetes Association (2003 Jul 1). Treatment of hypertension in adults with diabetes. Clin. Diabetes.

[17] WHO. HEARTS (2020). https://www.who.int/publications/i/item/9789240001367.

[18] WHO (2020). https://www.who.int/publications/i/item/who-package-of-essential-noncommunicable-(pen)-disease-interventions-for-primary-health-care.

[19] Gwatidzo S.D., Stewart Williams J. (2017 Jan 11). Diabetes mellitus medication use and catastrophic healthcare expenditure among adults aged 50+ years in China and India: results from the WHO study on global ageing and adult health (SAGE). BMC Geriatr..

[20] Zhang X., Xu Q., Guo X., Jing Z., Sun L., Li J. (2020 Apr 22). Catastrophic health expenditure: a comparative study between hypertensive patients with and without complication in rural Shandong, China. BMC Public Health.

[21] Zarei L., Moradi N., Peiravian F., Hatami-Mazinani N., Mahi-Birjand M., Arabloo J. (2022 Dec 29). Catastrophic pharmaceutical expenditure in patients with type 2 diabetes in Iran. Int. J. Equity Health.

[22] Devine J.W., Lim D., Lugo A., Farley J.F. (2023 Oct). Prevalence and patterns of catastrophic spending for antidiabetic medication in 2020. J. Manag. Care Spec. Pharm..

[23] Geldsetzer P., Manne-Goehler J., Marcus M.-E., Ebert C., Zhumadilov Z., Wesseh C.S. (2019 Aug). The state of hypertension care in 44 low-income and middle-income countries: a cross-sectional study of nationally representative individual-level data from 1.1 million adults. Lancet.

[24] Mohanty S.K., Pedgaonkar S.P., Upadhyay A.K., Kämpfen F., Shekhar P., Mishra R.S. (2021 Aug 24). Awareness, treatment, and control of hypertension in adults aged 45 years and over and their spouses in India: a nationally representative cross-sectional study. PLoS Med..

[25] Abalos J.B., Saito Y., Ramos M.A., Cruz G.T. (2023 Jun 28). Prevalence, awareness, treatment, and control of hypertension among older adults in the Philippines. J. Gerontol.: Series A.

[26] LaMonica L.C., McGarvey S.T., Rivara A.C., Sweetman C.A., Naseri T., Reupena M.S. (2022 Jan). Cascades of diabetes and hypertension care in Samoa: identifying gaps in the diagnosis, treatment, and control continuum – a cross-sectional study. Lancet Reg. Health West. Pac..

[27] Xiong S., Jiang W., Wang Y., Hu C., Yang J., Bao M. (2024 Apr). Using routinely collected data to determine care cascades of hypertension and type-2 diabetes management in China: a cross-sectional study. Lancet Reg. Health West. Pac..

[28] Stein D.T., Reitsma M.B., Geldsetzer P., Agoudavi K., Aryal K.K., Bahendeka S. (2024 Jan 26). Hypertension care cascades and reducing inequities in cardiovascular disease in low- and middle-income countries. Nat. Med..

[29] Frieden T.R., Garg R., Banigbe B., Choudhury S., Ogbureke N., Duguma D. (2025 Mar). Unlocking health equity by eliminating copayments for essential antihypertensive medications. eClinicalMedicine.

[30] Department of Health, Republic of the Philippines (DOH) (2016).

[31] DOH. Administrative Order No. 2012-0029 (2012 December 04).

[32] Philippine Statistics Authority (PSA) and ICF (2023).

[33] Capuno J., Kraft A.D., O'Donnell O. (2021). Effectiveness of clinic-based cardiovascular disease prevention: a randomized encouragement design experiment in the Philippines. Soc. Sci. Med..

[34] Lambojon K., Chang J., Saeed A., Hayat K., Li P., Jiang M. (2020 Jul 21). Prices, availability and affordability of medicines with value-added tax exemption: a cross-sectional survey in the Philippines. Int. J. Environ. Res. Publ. Health.

[35] Gutierrez M.M., Sakulbumrungsil R. (2021 Oct 1). Factors associated with medication adherence of hypertensive patients in the Philippines: a systematic review. J. Clin. Hypertens..

[36] Cando L.F., Quebral E.P., Ong E.P., Catral C.D., Relador R.J., Velasco A.J. (2024 Feb). Current status of diabetes mellitus care and management in the Philippines. Diabetes Metab. Syndr. Clin. Res. Rev..

[37] Cruz G.T., Cruz C.J.P., Saito Y. (2019). Ageing and Health in the Philippines.

[38] Filmer D., Pritchett L.H. (2001 Feb 1). Estimating wealth effects without expenditure data—or tears: an application to educational enrollments in states of India. Demography.

[39] Verbrugge L.M. (2016 Sept 1). Disability experience and measurement. J. Aging Health.

[40] Van Oyen H., Bogaert P., Yokota R.T., Berger N. (2018 May 28). Measuring disability: a systematic review of the validity and reliability of the global activity limitations indicator (Gali). Arch. Public Health.

[41] Pfeiffer E. (1975 Oct). A short portable mental status questionnaire for the assessment of organic brain deficit in elderly patients. J. Am. Geriatr. Soc..

[42] Zhou B., Rayner A.W., Gregg E.W., Sheffer K.E. (2024). Worldwide trends in diabetes prevalence and treatment from 1990 to 2022: a pooled analysis of 1108 population representative studies with 141 million participants. Lancet.

[43] Kraft A.D., Capuno J.J., Calicdan K.G., Cruz G.T., O'Donnell O. (2024 Sept). Missed opportunities for hypertension screening of older people in the Philippines: Cross-sectional analysis of nationally representative individual-level data. Lancet Reg. Health West. Pac..

[44] Uiep V.G., Uy J., Casas L.D. (2020). Primary health care for noncommunicable diseases in the Philippines. PIDS Discussion Paper Series.

[45] World Health Organization (2021).

[46] World Health Organization (2025).

[47] Mullainathan S., Shafir E. (2014).

[48] Schilbach F., Schofield H., Mullainathan S. (2016 May 1). The psychological lives of the poor. Am. Econ. Rev..

