# Supplementary material for: Inequality in prevalence of unmedicated hypertension or diabetes among older Filipinos: analysis of nationally representative survey data
Source: Int J Cardiol Cardiovasc Risk Prev. 2026 Mar 2;29:200617. doi: 10.1016/j.ijcrp.2026.200617 (PMC12989984; doi:10.1016/j.ijcrp.2026.200617)
Supplement: Multimedia component 1 [file mmc1.docx]

**Supplementary Material**

**Inequality in prevalence of unmedicated hypertension or diabetes among older Filipinos:**

**analysis of nationally representative survey data**

| **Table S1**. Indicators included in principal components analysis and their weights used to construct the wealth index | | | | |
| --- | --- | --- | --- | --- |
|  | Mean | SD | Weight | Weight/SD |
| Household owns (yes = 1, no = 0) |  |  |  |  |
| Car | 0.047 | 0.211 | 0.043 | 0.202 |
| Motorcycle/tricycle | 0.238 | 0.426 | 0.053 | 0.124 |
| Motorized boat/banca | 0.031 | 0.174 | 0.000 | 0.003 |
| Air conditioner | 0.085 | 0.278 | 0.059 | 0.212 |
| Washing machine | 0.340 | 0.474 | 0.083 | 0.175 |
| Stove | 0.173 | 0.378 | 0.057 | 0.149 |
| Refrigerator | 0.391 | 0.488 | 0.088 | 0.181 |
| Computer | 0.107 | 0.309 | 0.060 | 0.196 |
| Cellular/mobile phone | 0.660 | 0.474 | 0.071 | 0.151 |
| Landline/wireless phone | 0.039 | 0.194 | 0.042 | 0.214 |
| Audio component/stereo set | 0.129 | 0.336 | 0.049 | 0.145 |
| Karaoke/videoke/Magic Sing | 0.062 | 0.241 | 0.035 | 0.144 |
| CD/VCD/DVD player | 0.206 | 0.404 | 0.052 | 0.129 |
| Television | 0.718 | 0.450 | 0.084 | 0.188 |
| Radio/cassette player | 0.353 | 0.478 | 0.022 | 0.046 |
| Household has electricity | 0.922 | 0.268 | 0.058 | 0.215 |
| Household has internet | 0.168 | 0.374 | 0.066 | 0.178 |
| House/lot ownership/tenure |  |  |  |  |
| Own house and lot | 0.665 | 0.472 | 0.042 | 0.088 |
| Own house, rent lot | 0.030 | 0.170 | 0.000 | 0.000 |
| Own house, rent-free lot with consent of owner | 0.187 | 0.390 | -0.048 | -0.123 |
| Own house, rent-free lot without consent of owner | 0.019 | 0.137 | -0.006 | -0.046 |
| Drinking water source |  |  |  |  |
| Piped into dwelling | 0.234 | 0.423 | -0.032 | -0.076 |
| Piped to yard/plot | 0.034 | 0.181 | -0.041 | -0.227 |
| Piped to neighbor | 0.038 | 0.191 | -0.058 | -0.306 |
| Piped to public tap/standpipe | 0.085 | 0.278 | -0.074 | -0.265 |
| Tubed well/borehole | 0.091 | 0.287 | -0.060 | -0.208 |
| Protected dug well/spring | 0.085 | 0.279 | -0.076 | -0.271 |
| Unprotected/Rainwater/Tanker | 0.018 | 0.132 | -0.030 | -0.230 |
| Refilling station/Bottled water | 0.416 | 0.493 | 0.000 | 0.000 |
| Other water source |  |  |  |  |
| Piped into dwelling | 0.553 | 0.497 | 0.000 | 0.000 |
| Piped to yard/plot | 0.053 | 0.224 | -0.057 | -0.255 |
| Piped to neighbor | 0.041 | 0.199 | -0.064 | -0.321 |
| Public taps/standpipe | 0.090 | 0.286 | -0.083 | -0.292 |
| Tubed well/borehole | 0.124 | 0.329 | -0.077 | -0.233 |
| Protected dug well/spring | 0.096 | 0.295 | -0.086 | -0.293 |
| Unprotected/Rainwater/Tanker | 0.043 | 0.203 | -0.060 | -0.294 |
| Household sanitation |  |  |  |  |
| Flush to piped sewer system | 0.008 | 0.086 | -0.013 | -0.145 |
| Flush to septic tank | 0.891 | 0.311 | 0.000 | 0.000 |
| Flush to pit latrine | 0.035 | 0.185 | -0.067 | -0.363 |
| Flush to somewhere else | 0.005 | 0.069 | -0.021 | -0.298 |
| Non-flush/no toilet | 0.061 | 0.239 | -0.109 | -0.456 |
| Roof |  |  |  |  |
| Strong/predominantly strong materials | 0.874 | 0.332 | 0.074 | 0.221 |
| Light/predominantly light materials | 0.116 | 0.321 | -0.071 | -0.221 |
| Salvaged/predominantly salvaged materials | 0.010 | 0.097 | -0.017 | -0.178 |
| Outer walls |  |  |  |  |
| Strong/predominantly strong materials | 0.700 | 0.458 | 0.095 | 0.208 |
| Light/predominantly light materials | 0.270 | 0.444 | -0.088 | -0.198 |
| Salvaged/predominantly salvaged materials | 0.028 | 0.166 | -0.027 | -0.162 |
| Floor |  |  |  |  |
| Natural flooring | 0.054 | 0.226 | -0.077 | -0.341 |
| Rudimentary flooring | 0.182 | 0.386 | -0.137 | -0.355 |
| Finished flooring - Ceramic tiles | 0.201 | 0.400 | 0.000 | 0.000 |
| Finished flooring - Cement | 0.540 | 0.498 | -0.065 | -0.130 |
| Finished flooring - Others | 0.024 | 0.152 | -0.025 | -0.164 |
| Natural flooring | 0.128 | 0.335 | 0.000 | 0.000 |
| Conditional cash transfer (4P) beneficiary | 0.054 | 0.226 | -0.077 | -0.341 |

| Table S2. Characteristics of full sample, aged 60+ years |  |  |  |
| --- | --- | --- | --- |
|  | N | (%) |  |
| Overall | 5,985 | (100.0) |  |
| Diagnosis |  |  |  |
| Neither | 2,895 | (51.1) |  |
| Hypertension only | 2,380 | (36.3) |  |
| Diabetes only | 220 | (3.4) |  |
| Both hypertension & diabetes | 490 | (9.2) |  |
| Wealth quintile |  |  |  |
| Poorest | 989 | (20.0) |  |
| Poorer | 1,264 | (20.1) |  |
| Middle | 1,243 | (20.1) |  |
| Richer | 1,292 | (19.8) |  |
| Richest | 1,197 | (20.0) |  |
| Education |  |  |  |
| ≤ Elementary | 4,004 | (73.1) |  |
| Intermediate | 1,401 | (19.0) |  |
| ≥ College | 580 | (7.9) |  |
| Sex |  |  |  |
| Females | 3,823 | (59.7) |  |
| Males | 2,162 | (40.3) |  |
| Age |  |  |  |
| 60-64 years | 1,189 | (35.0) |  |
| 65-69 years | 961 | (27.8) |  |
| 70-74 years | 1,381 | (15.1) |  |
| 75-79 years | 962 | (10.8) |  |
| 80+ years | 1,492 | (11.2) |  |
| Location |  |  |  |
| Urban | 2,592 | (41.9) |  |
| Rural | 3,393 | (58.1) |  |
| Living arrangement |  |  |  |
| with children | 3,591 | (60.2) |  |
| Alone | 790 | (13.5) |  |
| with spouse only | 550 | (9.3) |  |
| with others | 1,054 | (17.0) |  |
| Working |  |  |  |
| Yes | 1,907 | (46.0) |  |
| No | 4,078 | (54.0) |  |
| Welfare recipient |  |  |  |
| Yes | 769 | (13.4) |  |
| No | 5,216 | (86.6) |  |
| Remittance income |  |  |  |
| Yes | 4,493 | (67.3) |  |
| No | 1,492 | (32.7) |  |
| Health insurance |  |  |  |
| Yes | 4,570 | (80.3) |  |
| No | 1,415 | (19.7) |  |
| Registered senior citizen |  |  |  |
| Yes | 5,305 | (89.1) |  |
| No | 680 | (10.9) |  |
| Forgone care |  |  |  |
| Yes | 1,597 | (29.0) |  |
| No | 4,388 | (71.0) |  |
| Blood pressure |  |  |  |
| High: SBP>=140 OR DBP>=90 | 3,113 | (51.4) |  |
| Normal: SBP<140 OR DBP<90 | 2,467 | (43.1) |  |
| Incomplete reading | 405 | (5.6) |  |
| Limited ADL |  |  |  |
| Yes | 1,415 | (21.7) |  |
| No | 4,570 | (78.3) |  |
| Cognitively impaired |  |  |  |
| Yes | 816 | (12.0) |  |
| No | 5,169 | (88.0) |  |
| Smoking |  |  |  |
| Non-smoker | 3,580 | (56.7) |  |
| Ex-smoker | 1,603 | (26.2) |  |
| Current smoker | 802 | (17.1) |  |
| Full sample used to estimate prevalence of diagnosed hypertension or diabetes.  Numbers are unweighted, while percentages are weighted. | | | |

| Table S3. Prevalence of diagnosed hypertension or diabetes, older (60+) Filipinos (N = 5985) | | | | | |
| --- | --- | --- | --- | --- | --- |
|  |  | % | 95% CI) | | P |
| Overall |  | 48.9 | (45.4, | 52.3) |  |
| Wealth quintile |  |  |  |  | <0.001 |
| Poorest |  | 34.2 | (27.2, | 42.0) |  |
| Poorer |  | 42.9 | (36.6, | 49.4) |  |
| Middle |  | 48.7 | (40.9, | 56.6) |  |
| Richer |  | 61.6 | (53.7, | 68.9) |  |
| Richest |  | 57.0 | (48.9, | 64.8) |  |
| Education |  |  |  |  | 0.001 |
| ≤ Elementary |  | 45.2 | (41.1, | 49.3) |  |
| Intermediate |  | 56.5 | (50.4, | 62.3) |  |
| ≥ College |  | 64.4 | (50.6, | 76.2) |  |
| Sex |  |  |  |  | <0.001 |
| Females |  | 53.0 | (48.5, | 57.4) |  |
| Males |  | 42.7 | (37.5, | 48.2) |  |
| Age |  |  |  |  | 0.118 |
| 60-64 years |  | 49.7 | (43.2, | 56.3) |  |
| 65-69 years |  | 42.9 | (35.6, | 50.5) |  |
| 70-74 years |  | 53.1 | (47.3, | 58.8) |  |
| 75-79 years |  | 52.9 | (44.5, | 61.2) |  |
| 80+ years |  | 51.4 | (46.0, | 56.7) |  |
| Location |  |  |  |  | 0.002 |
| Urban |  | 55.3 | (49.2, | 61.2) |  |
| Rural |  | 44.2 | (40.3, | 48.2) |  |
| Living arrangement |  |  |  |  | 0.065 |
| with children |  | 47.8 | (43.3, | 52.3) |  |
| Alone |  | 41.0 | (31.7, | 50.9) |  |
| with spouse only |  | 55.1 | (45.9, | 64.1) |  |
| with others |  | 55.4 | (47.6, | 63.0) |  |
| Working |  |  |  |  | <0.001 |
| Yes |  | 41.2 | (35.7, | 46.8) |  |
| No |  | 55.4 | (51.2, | 59.5) |  |
| Welfare recipient |  |  |  |  | <0.001 |
| Yes |  | 37.3 | (29.9, | 45.4) |  |
| No |  | 50.6 | (46.9, | 54.4) |  |
| Remittance income |  |  |  |  | 0.091 |
| Yes |  | 51.0 | (47.1, | 54.8) |  |
| No |  | 44.5 | (37.7, | 51.5) |  |
| Health insurance |  |  |  |  | 0.901 |
| Yes |  | 48.8 | (45.0, | 52.6) |  |
| No |  | 49.2 | (41.2, | 57.2) |  |
| Registered senior citizen |  |  |  |  | 0.007 |
| Yes |  | 50.4 | (46.7, | 54.1) |  |
| No |  | 36.3 | (27.6, | 45.8) |  |
| Forgone care |  |  |  |  | 0.421 |
| Yes |  | 46.7 | (40.9, | 52.6) |  |
| No |  | 49.7 | (45.5, | 54.0) |  |
| Blood pressure |  |  |  |  | 0.003 |
| High: SBP>=140 OR DBP>=90 |  | 56.1 | (51.3, | 60.8) |  |
| Normal: SBP<140 OR DBP<90 |  | 40.4 | (35.1, | 45.9) |  |
| Incomplete reading |  | 47.7 | (36.1, | 59.6) |  |
| Restricted ≥ 1 ADL |  |  |  |  | 0.002 |
| Yes |  | 61.7 | (54.3, | 68.6) |  |
| No |  | 45.3 | (41.5, | 49.2) |  |
| Cognitively impaired |  |  |  |  | 0.047 |
| Yes |  | 40.0 | (31.7, | 49.0) |  |
| No |  | 50.1 | (46.3, | 53.8) |  |
| Smoking |  |  |  |  | 0.001 |
| Non-smoker |  | 53.9 | (49.3, | 58.5) |  |
| Ex-smoker |  | 47.5 | (41.2, | 54.0) |  |
| Current smoker |  | 34.0 | (26.8, | 42.2) |  |
| P (values) from z tests of equal proportions between 2 groups and chi-square tests of independence for > 2 groups. | | | | | |

| Table S4. Unmedicated risk difference (RD) by covariates, older Filipinos diagnosed with hypertension or diabetes and with high blood pressure (N = 1827) | | | | |  |
| --- | --- | --- | --- | --- | --- |
|  | RD (pp) | (95% CI) | P | |  |
| Diagnosis (ref. hypertension only) |  |  |  | |  |
| Diabetes only | -2.3 | (-15.1, 10.4) | 0.717 | |  |
| Both HTN and diabetes | 3.4 | (-5.1, 11.9) | 0.430 | |  |
| Wealth quintile (ref. Richest) |  |  |  | |  |
| Poorest | 23.7 | (8.3, 39.1) | 0.003 | |  |
| Poorer | 8.9 | (-3.1, 20.9) | 0.144 | |  |
| Middle | 9.0 | (-1.5, 19.5) | 0.093 | |  |
| Richer | 4.6 | (-4.4, 13.5) | 0.314 | |  |
| Educational attainment (ref. ≥ College) |  |  |  | |  |
| ≤ Elementary | 10.2 | (0.1, 20.4) | 0.048 | |  |
| Intermediate | 10.3 | (0.1, 20.5) | 0.048 | |  |
| Sex (ref. male) |  |  |  | |  |
| Females | 0.2 | (-9.0, 9.4) | 0.958 | |  |
| Age (ref. 80+) |  |  |  | |  |
| 60-64 years | -8.7 | (-21.2, 3.7) | 0.168 | |  |
| 65-69 years | 4.5 | (-9.7, 18.7) | 0.531 | |  |
| 70-74 years | -8.9 | (-21.4, 3.7) | 0.166 | |  |
| 75-79 years | -8.0 | (-21.3, 5.4) | 0.240 | |  |
| Location (ref. Urban) | |  |  |  | |
| Rural | | 4.2 | (-5.1, 13.4) | 0.374 | |
| Living (ref. with others) | |  |  |  | |
| with children | | -3.0 | (-11.5, 5.5) | 0.488 | |
| Alone | | -3.3 | (-15.1, 8.6) | 0.587 | |
| with spouse only | | 1.5 | (-12.6, 15.6) | 0.836 | |
| Working (ref. No) | |  |  |  | |
| Currently working | | 4.9 | (-2.9, 12.7) | 0.215 | |
| Welfare recipient (ref. Yes) | |  |  |  | |
| No | | 4.5 | (-5.5, 14.4) | 0.376 | |
| Remittance income (ref. Yes) | |  |  |  | |
| No | | 5.9 | (-2.4, 14.3) | 0.162 | |
| Health insurance (ref. Yes) | |  |  |  | |
| No | | 6.0 | (-2.4, 14.4) | 0.162 | |
| Senior citizen registration (ref. Yes) | |  |  |  | |
| No | | 24.8 | (13.5, 36.1) | <0.001 | |
| Forgone care (ref. No) |  |  |  | |  |
| Yes | -1.4 | (-8.5, 5.6) | 0.685 | |  |
| Limited ADL (ref. No) |  |  |  | |  |
| Yes | -6.2 | (-13.2, 0.8) | 0.083 | |  |
| Cognitively impaired (ref. No) |  |  |  | |  |
| Yes | 2.8 | (-8.4, 14.0) | 0.617 | |  |
| Smoking (ref. Non-smoker) |  |  |  | |  |
| Ex-smoker | 5.3 | (-4.8, 15.3) | 0.301 | |  |
| Current smoker | 10.6 | (-2.3, 23.5) | 0.107 | |  |
| Note. Percentage point (pp) risk difference in probability of being unmedicated compared with reference category. Province/municipality fixed effects also included in probit model. | | | | | |
