# Supplementary material for: Inequality in prevalence of unmedicated hypertension or diabetes among older Filipinos: analysis of nationally representative survey data
Source: Int J Cardiol Cardiovasc Risk Prev. 2026 Mar 2;29:200617. doi: 10.1016/j.ijcrp.2026.200617 (PMC12989984; doi:10.1016/j.ijcrp.2026.200617)
Supplement: Multimedia component 2 [file mmc2.docx]

**Supplementary Checklist S1.** STROBE Statement for cross-sectional study – Inequality in unmedicated hypertension or diabetes among older Filipinos: nationally representative survey data.

|  | **Item No** | **Recommendation** | **Page No.** | **Relevant text from manuscript** | |
| --- | --- | --- | --- | --- | --- |
| **Title and abstract** | 1 | (*a*) Indicate the study’s design with a commonly used term in the title or the abstract | 1 | *analysis of nationally representative individual-level data* | |
|  |  | (*b*) Provide in the abstract an informative and balanced summary of what was done and what was found | 2 | *Methods* and *Findings* sections of *Abstract* | |
| **Introduction** | | |  | |  |
| Background/rationale | 2 | Explain the scientific background and rationale for the investigation being reported | *3-4* | Text of *Introduction* | |
| Objectives | 3 | State specific objectives, including any prespecified hypotheses | 4 | *This study aimed to estimate inequality in the prevalence of unmedicated hypertension or diabetes among older Filipinos diagnosed with either condition.* | |
| **Methods** | | |  | |  |
| Study design | 4 | Present key elements of study design early in the paper | 2, 4-7 | *Title* and *Methods* section of *Abstract.*  *Methods, Data*, *Measurements, Statistical Analysis.* | |
| Setting | 5 | Describe the setting, locations, and relevant dates, including periods of recruitment, exposure, follow-up, and data collection | *4-5* | *We used data from the first wave of the Longitudinal Study on Ageing and Health in the Philippines (LSAHP), conducted from October 2018 to February 2019.* | |
| Participants | 6 | (*a*) Give the eligibility criteria, and the sources and methods of selection of participants | 4, 6 | *representative of the population aged 60 years and older (60+)*  *The analysis sample included participants categorized as diagnosed with hypertension, diabetes or both.* | |
| Variables | 7 | Clearly define all outcomes, exposures, predictors, potential confounders, and effect modifiers. Give diagnostic criteria, if applicable | 5-7 | *Methods: Measurements, Statistical Analysis* | |
| Data sources/ measurement | 8* | For each variable of interest, give sources of data and details of methods of assessment (measurement). Describe comparability of assessment methods if there is more than one group | 5-7 | *Methods: Measurements, Statistical Analysis* | |
| Bias | 9 | Describe any efforts to address potential sources of bias | 6-7 | *We adjusted for age and sex using the age-sex composition of the analysis sample as the reference…*  *We estimated the fully adjusted difference in the risk of being undiagnosed between each category and a reference category of each covariate…*  *Sample weights were applied in all analyses...* | |
| Study size | 10 | Explain how the study size was arrived at | 20 | *Figure 1* | |
| Quantitative variables | 11 | Explain how quantitative variables were handled in the analyses. If applicable, describe which groupings were chosen and why | 5-7 | *Methods: Measurements, Statistical Analysis* | |
| Statistical methods | 12 | (*a*) Describe all statistical methods, including those used to control for confounding | *6-7* | *We adjusted for age and sex using the age-sex composition of the analysis sample as the reference…*  *We estimated the fully adjusted difference in the risk of being undiagnosed between each category and a reference category of each covariate…* | |
|  |  | (*b*) Describe any methods used to examine subgroups and interactions | *6-7* | See text referred to immediately above. | |
|  |  | (*c*) Explain how missing data were addressed | *6* | *… blood pressure (BP) was measured three times (with a one-minute gap). We used the average from the last two readings to distinguish between three categories of blood pressure: high…, …normal and …incomplete (< 3 BP measurements taken).*  *There were no missing data on wealth and covariates.* | |
|  |  | (*d*) If applicable, describe analytical methods taking account of sampling strategy | 6-7 | *Methods: Statistical Analysis* | |
|  |  | (*e*) Describe any sensitivity analyses | SM | *Table S4. Unmedicated risk differences (RD) by covariates, older Filipinos diagnosed with hypertension or diabetes and with high blood pressure* | |
| **Results** | | |  | |  |
| Participants | 13* | (a) Report numbers of individuals at each stage of study—eg numbers potentially eligible, examined for eligibility, confirmed eligible, included in the study, completing follow-up, and analysed | 19 | *Figure 1* | |
|  |  | (b) Give reasons for non-participation at each stage | 19 | *Figure 1* | |
|  |  | (c) Consider use of a flow diagram | 19 | *Figure 1* | |
| Descriptive data | 14* | (a) Give characteristics of study participants (eg demographic, clinical, social) and information on exposures and potential confounders | 20 | *Table 1* | |
|  |  | (b) Indicate number of participants with missing data for each variable of interest | 19 | *Figure 1* | |
| Outcome data | 15* | Report numbers of outcome events or summary measures | *20-21* | *Table 1, Table 2* | |
| Main results | 16 | (*a*) Give unadjusted estimates and, if applicable, confounder-adjusted estimates and their precision (eg, 95% confidence interval). Make clear which confounders were adjusted for and why they were included | *20-22* | *Tables 1-3* | |
|  |  | (*b*) Report category boundaries when continuous variables were categorized | 20 | *Table 1* | |
|  |  | (*c*) If relevant, consider translating estimates of relative risk into absolute risk for a meaningful time period | 22 | *Tables 3* | |
| Other analyses | 17 | Report other analyses done—eg analyses of subgroups and interactions, and sensitivity analyses | SM | *Table S4* | |
| **Discussion** | | |  | |  |
| Key results | 18 | Summarise key results with reference to study objectives | *9, 10* | *Propensity to be unmedicated decreases steeply with increasing wealth (P < 0.001): an estimated 57.8% (43.2, 71.5) of those in the poorest fifth of older Filipinos and who were diagnosed with either condition are unmedicated, compared with 13.2% (95% CI: 9.0, 18.7) of the richest fifth.*  *We estimated that those diagnosed with diabetes only are 22.7 pp (6.5, 38.8) more likely to be unmedicated than those diagnosed with hypertension only, with adjustment for all covariates. After that adjustment, the wealth gradient in the propensity to be unmedicated remained clear – the poorest fifth were estimated to be 28.3 pp (13.6, 43.0) more likely to go unmedicated than the richest fifth…*  *Our finding that almost one third of older Filipinos diagnosed with hypertension or diabetes are unmedicated indicates a very large gap in management of principal risk factors for CVD….* | |
| Limitations | 19 | Discuss limitations of the study, taking into account sources of potential bias or imprecision. Discuss both direction and magnitude of any potential bias | *13-14* | *There are several study limitations. First, diagnosis was self-reported…. Second, we focused on treatment through medication and, due to data constraints, ignored medical advice on lifestyle modification…. Third, we did not examine consequences of unmedicated hypertension and diabetes measured by uncontrolled blood pressure and blood glucose, respectively…. Fourth, we relied on a binary indicator of taking medication or not because there were no data on whether medications* *were taken at the prescribed frequency and dose…. Finally, the data do not provide information on the duration for which a diagnosed condition has gone unmedicated….* | |
| Interpretation | 20 | Give a cautious overall interpretation of results considering objectives, limitations, multiplicity of analyses, results from similar studies, and other relevant evidence | 14 | *… we provide evidence that a large proportion of older Filipinos diagnosed with hypertension or diabetes are living with their condition without management through pharmacotherapy and there is substantial inequality in unmedicated hypertension and diabetes to the disadvantage of poorer older persons. Both main findings underscore the need to strengthen the current programme of Hypertension and Diabetes Clubs in the Philippines, and they provide an important lesson to other countries: entitlement to free maintenance medication at public clinics is not sufficient to reduce substantial inequalities in treatment of (diagnosed) chronic conditions.* | |
| Generalisability | 21 | Discuss the generalisability (external validity) of the study results | 14 | *…and they provide an important lesson to other countries: entitlement to free maintenance medication at public clinics is not sufficient to reduce substantial inequalities in treatment of (diagnosed) chronic conditions.* | |
| **Other information** | | |  | |  |
| Funding | 22 | Give the source of funding and the role of the funders for the present study and, if applicable, for the original study on which the present article is based | *8, 14* | *Role of Funding Source*,  *Funding Source* | |
